# Supplementary material for: Drift Algal Accumulation in Ice Scour Pits Provides an Underestimated Ecological Subsidy in a Novel Antarctic Soft-Sediment Habitat
Source: Biology (Basel). 2023 Jan 13;12(1):128. doi: 10.3390/biology12010128 (PMC9855796; doi:10.3390/biology12010128)
Supplement: Supplementary file 1 [file biology-12-00128-s001.zip › biology-2083176-supplementary.pdf]

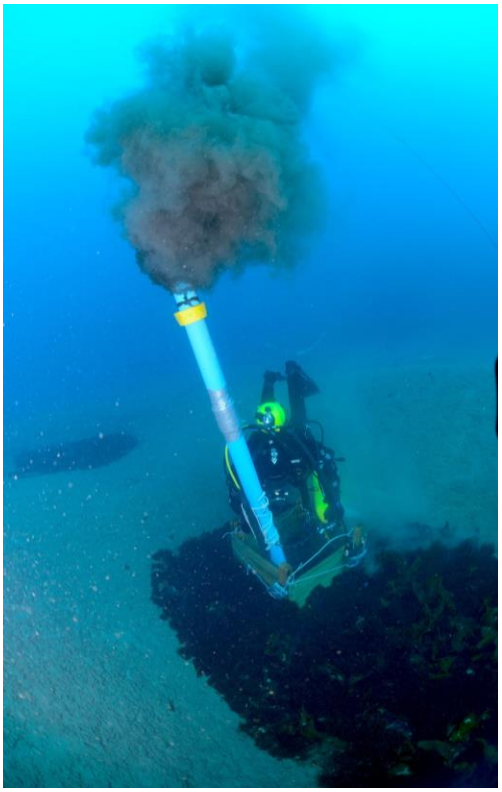

Figure S1: Suction sampling of 0.25-m^2 quadrat using an airlift device manipulated by a SCUBA diver.
